# Wearable Technology in Cardiology: Advancements, Applications, and Future Prospects

**DOI:** 10.31083/RCM39025

**Published:** 2025-06-27

**Authors:** Nihar Jena, Prabhat Singh, Deepak Chandramohan, Hari N. Garapati, Jyotsna Gummadi, Maneeth Mylavarapu, Bushra Firdous Shaik, Athmananda Nanjundappa, Dinesh Reddy Apala, Christian Toquica, Boney Lapsiwala, Prathap Kumar Simhadri

**Affiliations:** ^1^Department of Interventional Cardiology, Marshall University, Huntington, WV 25504, USA; ^2^Department of Nephrology, Kidney Specialists of South Texas, Corpus Christi, TX 78413, USA; ^3^Department of Nephrology, University of Alabama at Birmingham, Birmingham, AL 35233, USA; ^4^Department of Nephrology, Montgomery Kidney Specialists, Montgomery, AL 36116, USA; ^5^Department of Medicine, MedStar Franklin Square Medical Center, Baltimore, MD 21237, USA; ^6^Department of Health Informatics Management, Baptist Health Walker Hospital, Jasper, AL 35501, USA; ^7^Department of Internal Medicine Hospitalist, Piedmont Macon, Macon, GA 31217, USA; ^8^Department of Interventional Cardiology, Oklahoma Heart Hospital/Mercy Hospital, Ardmore, OK 73401, USA; ^9^Department of Cardiology, Trinity Health Oakland, Pontiac, MI 44405, USA; ^10^Department of Internal Medicine, Medical City Arlington, Arlington, TX 76015, USA; ^11^Department of Nephrology, Advent Health/FSU College of Medicine, Daytona Beach, FL 32117, USA

**Keywords:** wearable technology, cardiac monitoring, artificial intelligence, digital health, remote patient monitoring, arrhythmia detection

## Abstract

As the use of wearable devices continues to expand, their integration into various aspects of healthcare becomes increasingly prevalent. Indeed, significant advancements have been made in the field of cardiology through the application of wearable technology to monitor heart rate, rhythm, and other biological signals. This review examines the various applications of wearable technology in cardiology, with the goal of improving patient care. We evaluate the accuracy and functionality of existing wearable electrocardiograms, defibrillators, blood pressure monitors, fitness trackers, activity trackers, and sleep trackers, including their roles in cardiac rehabilitation. Furthermore, we highlight the significant advancements in wearable electrocardiograms, demonstrating their accuracy comparable to that of traditional monitoring devices, as shown by studies such as the Apple Heart Study and the Fitbit Heart Study. Recent research suggests that wearable electrocardiograms are comparable to conventional monitoring devices in terms of performance and can help reduce healthcare costs. However, as technological improvements continue to evolve, challenges related to accessibility, patient privacy, and the need for improved accuracy are also emerging. This review highlights recent advancements that aim to address these challenges. Nonetheless, further research is crucial to critically assess and identify shortcomings, as wearable devices possess significant potential to enhance cardiovascular and overall health.

## 1. Introduction

The utilization of monitoring devices for performance assessment has gained traction among athletes and endurance trainers. With the progress in medical science and technology, wearable devices are increasingly being embraced by practicing clinicians and the general population for health and fitness monitoring. These devices come in various forms, including smartwatches, bands, rings, and patches, and employ different mobile sensors that allow integration with the human interface to identify biosignals using complex proprietary software algorithms to analyze the data. Companies like Apple (Apple inc., Cupertino, CA, USA), Fitbit (Fitbit Inc., San Francisco, CA), and Garmin (Garmin Ltd., Olathe, KS) offer comparable products with similar features. All rely on optical photoplethysmography and electrocardiogram (ECG) to trace various health parameters.

This review aims to provide a comprehensive understanding of the rapidly evolving field of wearable technology in cardiology. We examine the diverse applications of these devices, tracing their historical development and critically evaluating their accuracy and functionality. We explore their expanding role in monitoring physical activity and sleep, emphasizing their impact on cardiac health and potential in rehabilitation. Furthermore, we address challenges related to data accuracy, privacy, and accessibility while discussing future directions such as AI integration and personalized medicine.

## 2. Early Developments in Wearable Technology

Norman Jefferis Holter is credited with pioneering ambulatory ECG and portable telemetry. During World War II, he was a senior physicist in the US Navy’s Bureau of Ships and contributed to the development of underwater operations. Holter initially developed an 84 lb device strapped like a backpack with short transmission capabilities and radio broadcasting of telemetry data. These efforts were made possible by the simultaneous progress in several fields, most notably, the development of reliable biopotential sensors for ECG recording, short-range radio communication for telemetry, and compact magnetic tape systems for storing continuous data. Together, these advancements laid the groundwork for truly ambulatory physiological monitoring. Subsequently, he collaborated with Del Mar Avionics, an aeronautic firm, to refine the device [1, 2]. Holter’s device is regarded as the foundation of modern wearable devices. The use of ambulatory electrocardiography was first reported in the Canadian Medical Association Journal in 1954. The first wearable heart monitors for endurance athletes were developed by a Finnish company named Polar Electro in 1978 and were made available for purchase at the consumer level in 1982, which was made possible by advancements in semiconductors [2].

Current-day developments have demonstrated increasing diversification in wearable technology applications. The emergence of smart textiles with embedded sensing capabilities in 2019 and advanced brain-machine interface initiatives such as Neuralink in 2020 suggest continuing evolution toward more sophisticated and integrated monitoring systems [3]. However, the early foundational roots of wearable technology dates back to the 13th century, marked by the earliest known record of wearable technology, eyeglasses, in 1268 [4].

The trajectory of wearable technology development demonstrates a remarkable progression from rudimentary body-worn tools to sophisticated biomedical monitoring systems. This evolution, which spanned multiple centuries, has been characterized by significant technological convergence and increasingly sophisticated integration of computational capabilities with physiological monitoring [5].

A major expansion in the foundations of wearable technology occurred in the early 20th century through Santos-Dumont’s development of the purpose-built wristwatch in 1907, which addressed specific operational requirements in aviation contexts. In 1993, Massachusetts Institute of Technology (MIT) researchers created the “Lizzy”, a wearable computer with a head-mounted display and one-handed keyboard, enabling real-time digital interaction. This marked a shift from single-function monitors to versatile, body-worn computing platforms [4]. The mid-20th century marked a paradigm shift toward computational integration through Thorp and Shannon’s pioneering development of a wearable computer system [6]. Although their device, designed for probability calculations in gaming applications, may appear tangential to medical monitoring, it set vital precedents for incorporating computational capabilities into portable, body-worn formats. Simultaneously, advancements in display technology, exemplified by Heilig’s 1960 patent for a head-mounted stereophonic television display, indicated emerging possibilities for information delivery in wearable formats.

The 1980s witnessed significant conceptual advancements, partly driven by cultural elements from science fiction media. These conceptual shifts were made technically feasible by progress in material engineering, particularly the development of anti-reflective coatings and compact sensors that made wearable displays and electronics more viable [6]. This period saw increased research focus on wearable displays and computational integration, establishing crucial groundwork for future developments in healthcare applications. Although advancements in mobile telephony primarily drove the technological landscape of the 1990s, it continued to make steady progress in wearable technology research, as evidenced by Defense Advanced Research Projects Agency (DARPA)’s “Wearables in 2005” workshop, which explored innovative applications in computerized clothing and body-mounted monitoring systems [4, 5, 6].

A critical inflection point occurred at the turn of the millennium with the introduction of Bluetooth wireless technology in 1999, establishing essential protocols for wireless data transmission in body-worn devices [4, 5, 6]. This period also coincided with significant developments in medical technology, including the release of digital pacemakers and early collaborations between technology and sportswear manufacturers, exemplified by the Nike-Apple partnership in fitness monitoring systems [4, 5, 6].

**Fig. 1.**
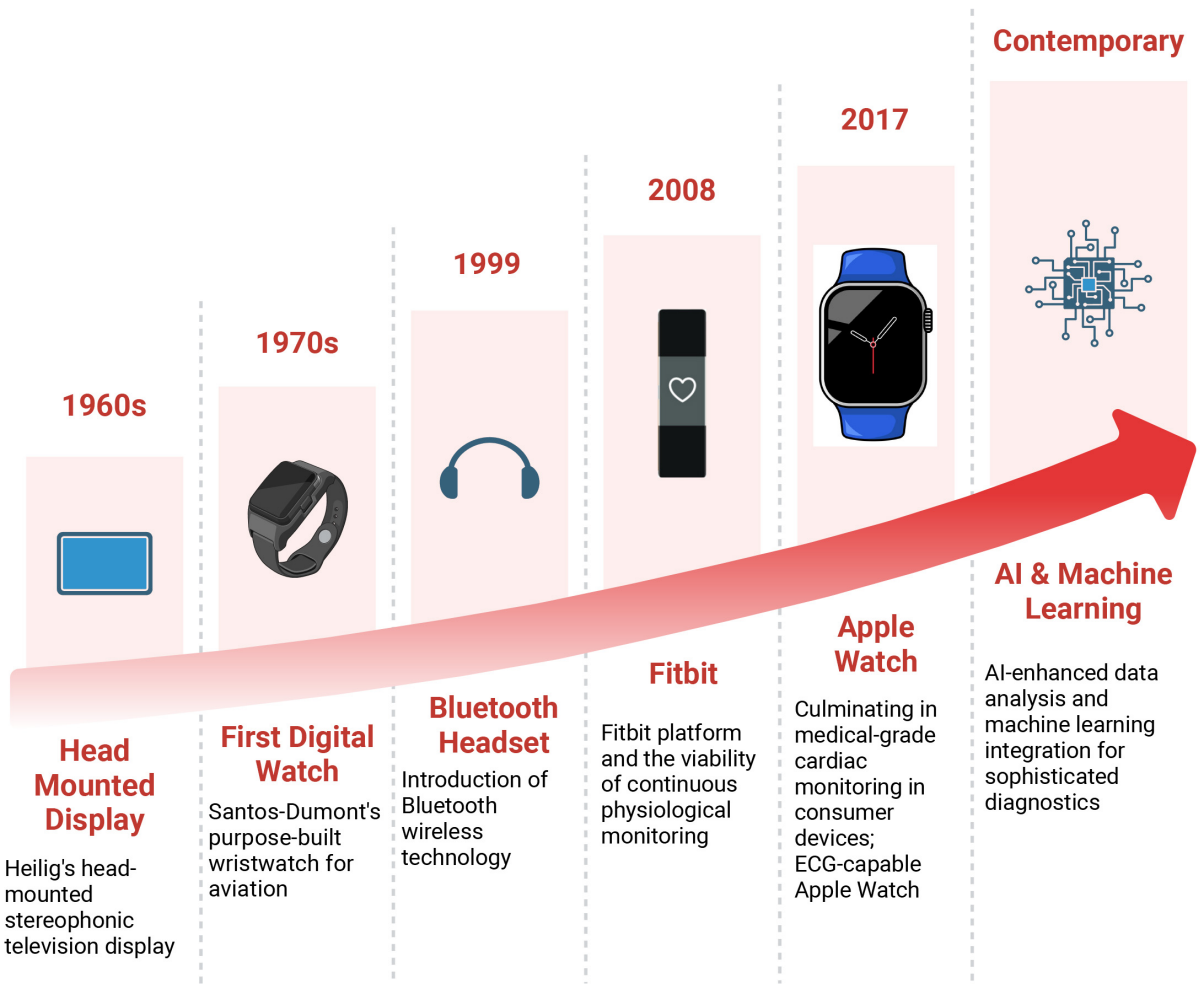
**Milestones of development in wearable devices that laid the foundation for modern cardiac monitoring**. AI, artificial intelligence.

The introduction of the Fitbit (Fitbit, Inc., San Francisco, USA) platform in 2008 represented a significant milestone in consumer-focused health monitoring, demonstrating the viability of continuous physiological monitoring through wearable technology. This development established foundational principles for subsequent innovations in cardiac monitoring systems [5]. The 2010s marked accelerated advancement in the field, culminating in the introduction of medical-grade cardiac monitoring capabilities in consumer devices, notably exemplified by the ECG-capable Apple Watch in 2017 [3]. Contemporary developments in artificial intelligence (AI), enhanced data analysis, and machine learning (ML) integration indicate the potential for increasingly sophisticated diagnostic and monitoring capabilities [3]. These innovations, paired with advances in miniaturized sensors and wireless communication, are driving a shift toward real-time cardiovascular monitoring and more personalized, proactive patient care [5]. Fig. 1 depicts the milestones of development in wearable devices.

## 3. Types of Wearable Devices in Cardiology

### 3.1 ECG Monitoring Devices

Several advancements in wearable cardiac devices have occurred since Holter first developed the ambulatory electrocardiography in 1947 [7]. However, the approval of a mobile cardiac outpatient telemetry (MCOT) system by CardioNet Inc. by the Food and Drug Administration (FDA) did not occur until 2002. This telemetry system utilized three-electrode lead sensors to transmit ECG waveforms continuously to a central monitoring system, which then sent the data to physicians [8]. Lately, the number of non-prescription devices has also increased. With many products available to consumers, advancements in sensors and other technologies have paved the way for further innovation. The global market for all wearable technology is estimated at USD 61.30 billion and is projected to grow at a compound annual growth rate of 14.6% from 2023 to 2030 [9]. Recently, the demand for remote monitoring has surged due to the rapid expansion of telemedicine in response to the coronavirus disease 2019 (COVID-19) pandemic [10, 11].

**Fig. 2.**
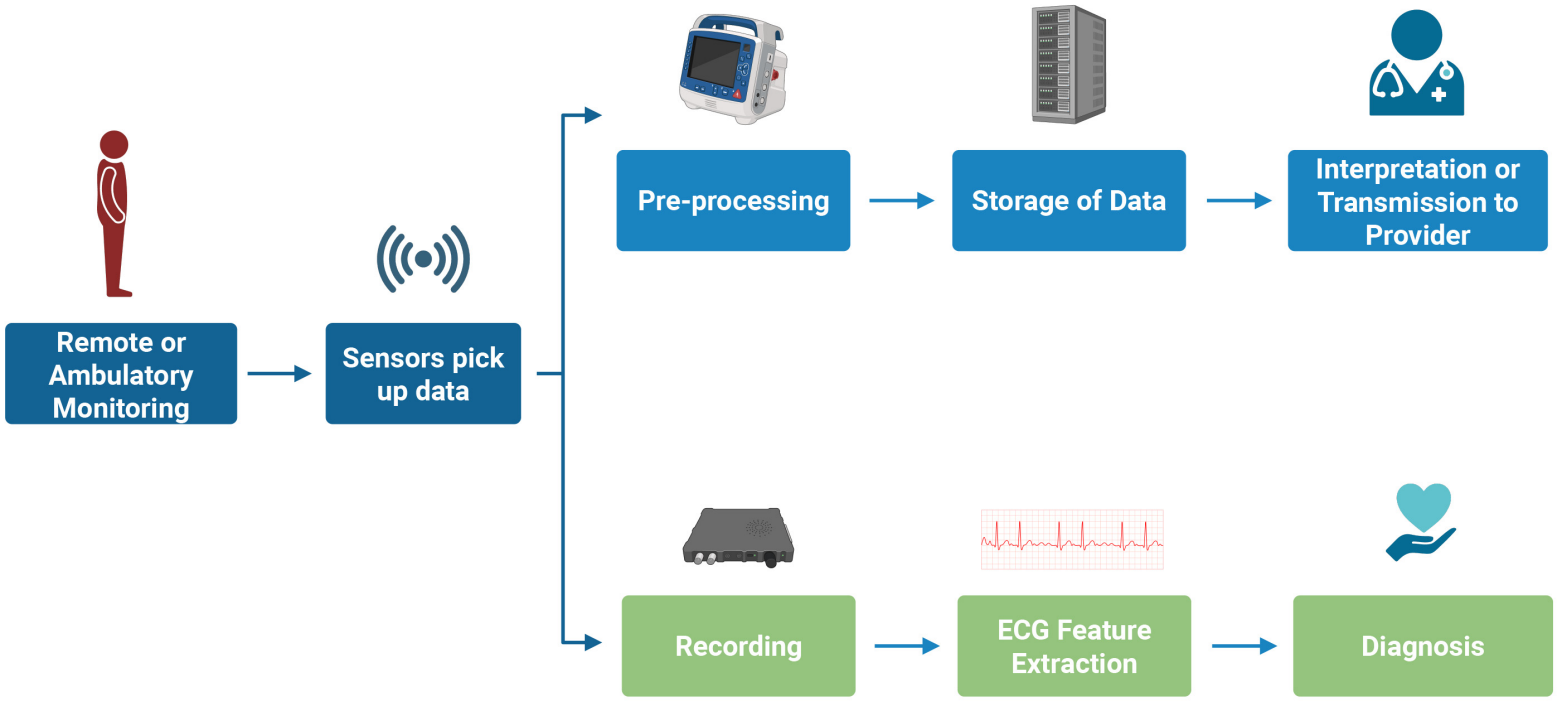
**Schematic overview of ECG monitoring highlighting the pathway from signal acquisition in wearable devices to clinically actionable interpretation**. ECG, electrocardiogram.

With technological advancements, the number of monitoring devices has increased, becoming an appealing area of research. Various types of wearable ECG devices exist, including patch devices that rely on contact with a surface for feature extraction, as well as contactless devices like smartwatches, shirts, and capacitive sensors embedded in patients’ hospital beds and wheelchairs [12, 13]. The development of dry/noncontact electrodes has made single-lead continuous ECG monitoring more convenient, although it may introduce more noise [13]. These single or three-lead devices can collect data passively or require active patient participation. The data is then transmitted in real time or stored in a central monitoring system for later analysis [12]. The collected data undergoes preprocessing using various techniques to eliminate noise, unwanted motion artifacts, and powerline interference [14]. Fig. 2 outlines the schematic representation of ECG monitoring. The currently available ambulatory monitoring equipment utilized in arrhythmia management can be categorized into continuous and noncontinuous monitoring systems. Most MCOT devices have the capability to monitor arrhythmias continuously using a single-lead ECG. They also allow for the analysis of long-term ECG data in real time and offline [10]. Devices used for automated detection of ECG patterns perform feature extraction to retrieve important representative features such as peak amplitudes, heart rate variability, and recognition of various segments and complexes to enable diagnosis [15].

#### 3.1.1 Types of Wearable ECG Devices

Several wearable ECG devices are broadly classified as:

∙ Mobile Cardiac Outpatient Telemetry (MCOT)

MCOT has been found to be superior in diagnosing arrhythmias (88% vs 75%) compared to loop recorder [16]. Typically, three lead sensors transmit the ECG data to a monitoring center via cell phone technology. In the case of an event, the ECG data is automatically transferred to the monitoring center, and certified technicians review this data, generate a report, and inform physicians accordingly. This type of monitor can be worn for up to 30 days [16].

∙ Wearable Heart Rhythm Monitors

Some monitors use photoplethysmography (PPG) to record pulse rates. Their algorithms are designed to detect significant changes in heart rate and, consequently, identify arrhythmia. Other devices utilize single or multiple lead ECGs to detect arrhythmias, such as atrial fibrillation. In addition to monitoring heart rate and rhythm, smartwatches can also track QT intervals [17]. These devices have also been employed to detect atrial fibrillation in patients with cryptogenic stroke. According to a meta-analysis, there was no statistically significant difference between smart wearable devices and conventional Holter monitoring in atrial fibrillation detection and cryptogenic stroke outcomes [18]. PPG relies on a technique that detects fluctuations in capillary bed volume in the skin based on heart rate variations. It uses a light source, typically green, to illuminate the skin and a photodetector to measure the reflected light [19]. Common brands of smartwatches and wristbands include Apple Watch, Fitbit, and Samsung smartwatches. The Apple Watch utilizes PPG technology, and newer models have also incorporated a single-lead ECG. The Apple Heart Study conducted in 2020 indicated that the Apple Watch has a positive predictive value of 84% for identifying atrial fibrillation [20]. Similarly, a Fitbit heart study demonstrated that the Fitbit smartwatch/band has a positive predictive value of 98% in detecting atrial fibrillation [21]. One limitation of smartwatches is their inability to perform continuous cardiac monitoring due to physical activity, which can interfere with the quality of ECG tracing.

∙ Smartphone Apps

Smartphone-integrated devices are gaining popularity due to their versatility and ease of use. Continuous smartphone ECG monitoring has demonstrated high sensitivity and specificity in detecting atrial fibrillation or atrial flutter. Most notably, they help detect arrhythmia recurrences post-ablation and prevent unnecessary hospital visits [22]. Similarly, smartphone-based event recorders have proven effective in real-time capturing symptomatic arrhythmias such as atrial fibrillation, atrial flutter, supraventricular arrhythmias, and atrial tachycardias [23]. These devices are cost-effective strategies for improving the detection of arrhythmias in inpatient and outpatient settings [23, 24]. Currently, FibriCheck (Qompium NV, Hasselt, Belgium) is the only smartphone-based app that the FDA has approved for rhythm monitoring. In a recent validation study, FibriCheck demonstrated a sensitivity of 100%, specificity of 98.9%, and overall accuracy of 99.2% in detecting AF [25]. Other non-FDA-approved apps, including Cardiio Rhythm Mobile, PULSE-SMART, and Preventicus, have also shown high sensitivity (94.2%) and specificity (95.8%), but relatively low positive predictive values (19.3%–37.5%) [23, 24].

∙ Wearable ECG Patches

They offer convenient, continuous cardiac monitoring that is less burdensome than traditional devices like Holter monitors. These patches store ECG tracings and can be worn for several days. However, data can only be analyzed after the patch is mailed. Such devices include Carnation Ambulatory Monitor (CAM, BardyDx, Washington, USA), ZioPatch WiPatch (iRhythm Technologies, CA, USA) (LifeSignals Inc, CA, USA), and VitalPatch (MediBioSense Ltd., Doncaster, UK). ZioPatch can be self-applied by the patient and records up to 14 days of continuous ECG monitoring. It is superior to the Holter monitor regarding atrial fibrillation detection after acute stroke [26]. Another benefit of patches is that they can detect rhythms other than atrial fibrillation, such as Supraventricular tachycardia.

∙ Smart Textile Garments

Garment-based cardiac monitoring has been developed to suit the needs of physically active individuals. In Japan, a company has developed a highly conductive fabric called Hitoe, which is used as a transmitter. Two electrodes are embedded in the t-shirt made of Hitoe fabric, and these wireless electrodes act as single-lead ECGs [27]. In 2019, 100 participants were used for two months to wear Hitoe fabric T-shirts. This study showed that these T-shirts were similar to other wearable devices in detecting atrial fibrillation [28].

Regarding purpose, not all wearable devices serve the same purpose. Medical-grade wearables such as Holter monitors, mobile cardiac telemetry systems, and FDA-cleared ECG patches are designed for diagnostic accuracy and clinical decision-making. In contrast, consumer-grade devices like smartwatches and fitness trackers, while useful for promoting personal health awareness, are not held to the same regulatory or performance standards. Although both categories may offer ECG monitoring capabilities, they differ substantially in accuracy, validation standards, and intended use. Recognizing these differences is essential when interpreting wearable data in clinical cardiology practice.

#### 3.1.2 Importance of Cardiac Health Monitoring

Atrial fibrillation, especially paroxysmal atrial fibrillation, is one of the arrhythmias that necessitate continuous cardiac monitoring, as untreated atrial fibrillation increases the risk of stroke. According to a study, approximately 25% of patients with acute ischemic stroke were found to have undiagnosed atrial fibrillation [29]. Therefore, early diagnosis is crucial. Continuous cardiac monitoring is superior in detecting arrhythmias compared to conventional twelve-lead ECGs because it allows for longer monitoring durations, which are limited to a snapshot in conventional twelve-lead ECGs. Traditionally, devices available for continuous cardiac monitoring include an event monitor (intermittent), an external loop recorder (which can monitor for longer durations, up to 30 days), and an implantable loop recorder that can record for up to 3 years, though this is an invasive procedure.

There is a growing demand for wearable ECG monitors that can detect arrhythmia at an early stage. With the advent of AI over the last decade, there has been tremendous improvement in the quality and accuracy of wearable cardiac monitors. This includes devices such as smartwatches, hand-held continuous ECG monitors, and others that allow for continuous cardiac monitoring in the comfort of one’s home. Wearable cardiac monitors are more convenient, relatively inexpensive, longer-lasting, and associated with better patient compliance. Still, they have a few shortcomings, such as suboptimal recording, low diagnostic yield, and inaccuracies in the diagnosis.

#### 3.1.3 Wearable ECG Devices in the United States

In the United States (USA), as of 2023, the Centers for Medicare and Medicaid Services (CMS) allows reimbursements for external ECG recording only for further evaluation of symptoms such as arrhythmias, chest pain, syncope, vertigo, palpitations, transient ischemic episodes, dyspnea, assessing the efficacy of antiarrhythmic therapy, monitoring myocardial infarction survivors with reduced ejection fraction, correlating chest pain with ST-segment changes in coronary artery disease and detecting recurrence of arrhythmias following ablation procedures. For reimbursements, ECG monitoring duration is divided into 48 hours, 48 hours up to 15 days, and long-term 30-day monitoring [30]. Table 1 outlines wearable ECG devices in the USA and their FDA status [31].

**Table 1.** **FDA status of wearable ECG devices**.

Type	Company/Brand	Product	FDA Status
Watches	Adidas	miCoach Fit Smart	NA
	Apple	Apple Watch series	A
	Biobeat	BB-613WP	A
	Fitbit	Flex, One, Charge, Sense, Versa, Luxe, Inspire	A
	Garmin	Epix Pro, Fenix 7 pro, Venu, Tactix 7	A
	Google	Pixel Watch	NA
	Huawei	Huawei Watch GT, Ultimate, Huawei Band	NA
	Karacus	DIONE, TRITON	NA
	Omron	HeartGuide	A
	Samsung	Galaxy Watch 3, 4, 5, 6	A
	SmartCardia	INYU	NA
	Tom Tom	TomTom Spark	NA
	Withings	Steel HR, Move, ScanWatch Horizon	A
Bands/Bracelets	AliveCor	Kardiaband	A
	BIOSTRAP	Armband HRM	NA
	Fitbit	Charge 4	A
	HEALBE	GoBe3	U
	Microsoft	Microsoft Band	NA
	MOCACARE	MOC cuff	A
	Under Armour	UA Band	NA
	Visi Mobile	The Visi Mobile System	A
	Xiaomi	Mi Smart Band 5	U
Patches	BardyDx	Zio Patch	A
	BioTelemetry	Bio Tel Heart	A
	Corventis Inc	Nuvant MCT	A
	Huinno	MEMO Patch	NA
	iRhythm	Zio Patch	A
	MediBioSense	MediBio Sense MBS HealthStream	A
	Preventice Solutions	BodyGuardian	A
	Samsung	S-Patch Ex	A
Clothes	HealthWatch Technologies (smart garments)	Master Caution	A
	Hexoskin (smart shirt)	Astroskin	NA
	Medtronic (chest strap)	Zephyr	A
	Polar (chest strap)	Polar H7 Strap	
	Sleeplay (smart sock)	Owlet Smart Sock 3	NA
	Spire Health Tag	Spire	NA
	Vivometrics (smart shirt)	The LifeShirt System	A
	Zoll (vest)	LifeVest	A
Miscellaneous	AliveCor (phone attachment)	KardiaMobile	A
	Personal Activity Intelligence (phone attachment)	PAI Health	U
	Motiv (ring)	Motiv Ring	NA
	Oura (finger ring)	Oura Ring	NA
	FreeWavz (smart earphones)	FreeWavz-Blue	U
	BioSensive Technologies (earrings)	Joule Earrings	NA
	SonoHealth	EK Graph	NA
	Jabra (headphones)	Sports Pulse Wireless Headphone	NA

*Legend: FDA, food and drug administration; A, approved; NA, not approved; U, unknown.

### 3.2 Wearable Defibrillators

Sudden cardiac death (SCD) accounts for >300,000 deaths in the United States annually [32]. First described in 1998 by Auricchio *et al*. [33], wearable defibrillators mark a significant advancement in cardiac care technology, providing a portable and non-invasive solution for patients at risk of SCD. The wearable cardioverter-defibrillator (WCD) is an external device that automatically detects and defibrillates ventricular tachycardia and ventricular fibrillation. The WCD also serves as an external loop recorder, continuously recording the patient’s heart rhythm and transmitting data on arrhythmias, including asystole and pauses [32, 34].

Regarding clinical efficacy, several prospective studies have reported successful defibrillations. The combined WEARIT/BIROAD study, which included patients with heart failure or post-MI complications, demonstrated successful defibrillation in most attempts [35]. The WEARIT-II registry provided further evidence of the WCD’s performance among a broader population of patients with various cardiac conditions. The VEST trial, the only randomized controlled trial to date, compared the WCD combined with guideline-directed medical therapy to guideline-directed medical therapy alone in post-MI patients with low ejection fraction, reporting a significant reduction in overall mortality for the WCD plus guideline-directed medical therapy group [35]. Supporting these findings, several observational studies have indicated mortality benefits associated with WCD use in specific high-risk groups [36, 37]. Major cardiology guidelines and consensus statements generally recommend the WCD as a bridging therapy for patients at high risk of SCD who are either not candidates for an implantable cardioverter-defibrillator (ICD) or have temporary contraindications to ICD implantation. Table 2 outlines the various guidelines for WCD use [38, 39, 40].

**Table 2.** **Guidelines recommendations for WCD use**.

Guideline source	Indication for WCD use	Class of recommendation	Level of evidence
2015 ESC Guidelines	Poor LV systolic function, risk of sudden arrhythmic death, not a candidate for ICD (bridge to transplant, bridge to transvenous implant, peripartum cardiomyopathy, active myocarditis, early post-MI arrhythmias)	IIb	C
	Selected patients 40 days post-MI (incomplete revascularization, pre-existing LVEF dysfunction, arrhythmias >48 h after ACS onset, polymorphic VT/VF)	IIb	C
	Bridging until recovery/ICD implantation in patients with inflammatory heart diseases, residual severe LV dysfunction, and/or ventricular electrical instability	IIa	C
2016 AHA Science Advisory	Clear indication for ICD but transient contraindication or interruption (e.g., infection)	IIa	C
	Bridge to more definitive therapy (e.g., cardiac transplantation)	IIa	C
	Concern about heightened SCD risk that may resolve (ischemic heart disease with recent revascularization, newly diagnosed NICM starting GDMT, secondary cardiomyopathy with treatable cause)	IIb	C
	Bridging therapy in situations with increased death risk where ICD reduces SCD but not overall survival (e.g., within 40 days of MI)	IIb	C
	Contraindicated when non-arrhythmic risk significantly exceeds arrhythmic risk, especially if survival <6 months	III	C
2017 AHA/ACC/HRS Guideline	ICD removal required (e.g., infection) in patients with history of SCA or sustained VA	IIa	B-NR
	Increased SCD risk, not ineligible for ICD (awaiting cardiac transplant, LVEF ≤35% within 40 days of MI, newly diagnosed NICM, revascularization within 90 days, myocarditis, secondary cardiomyopathy, systemic infection)	IIb	B-NR

*Legend: ACC, american college of cardiology; ACS, acute coronary syndrome; AHA, american heart association; ESC, european society of cardiology; GDMT, guideline-directed medical therapy; HRS, heart rhythm society; ICD, implantable cardioverter-defibrillator; LV, left ventricular; LVEF, left ventricular ejection fraction; MI, myocardial infarction; NICM, non-ischemic cardiomyopathy; NR, not randomized; SCA, sudden cardiac arrest; SCD, sudden cardiac death; VA, ventricular arrhythmia; VT/VF, ventricular tachycardia/ventricular fibrillation; WCD, wearable cardioverter-defibrillator.

### 3.3 Wearable Blood Pressure Devices

Hypertension (HTN) continues to remain an underdiagnosed and undertreated condition, and it is the most common cause of cardiovascular disease. According to CDC estimates, in 2023, 119.9 million, nearly half of US adults (48.1%), had high blood pressure (BP), and an estimated one billion people worldwide have HTN [40]. Only 22.5% of them have their HTN under control in the United States [41]. Annual direct medical expenses associated with HTN management are estimated at 131 billion USD in the United States [42]. Measuring BP remains the most essential procedure in clinical practice. However, according to an editorial by Norman Kaplan published in the American Journal of Hypertension in 1998, it is also the most carelessly performed procedure [43]. This observation still holds true even today.

Traditional BP monitoring involves inflating a cuff and measuring the BP using auscultatory and oscillatory methods. These methods cannot provide continuous BP monitoring and are at a higher risk for errors (i.e., by the use of non properly sized cuff or poor measurement technique). Ambulatory BP monitoring (ABPM) and home BP monitoring (HBPM) methods have been shown to correlate more closely with cardiovascular mortality than traditional BP monitoring [44]. However, they have limitations, as ABPM devices can be uncomfortable to wear and do not provide continuous BP monitoring.

BP is variable beat-to-beat and day-to-day and experiences seasonal shifts (higher peaks are noticed during exercise in winter). BP peaks based on various external environmental triggers, such as exercise, temperature, sleep apnea, and stress, are associated with increased cardiovascular events, especially in patients with increased arterial stiffness and decreased arterial absorbance [45]. Patients with excessive morning BP surges have an increased risk of developing hypertension-mediated organ damage, stroke, and cerebral hemorrhage. Nocturnal non-dipping and nocturnal hypertension are associated with cardiovascular events in both normotensive and hypertensive patients [45].

Wearable BP monitoring devices represent a promising future in frequent BP monitoring, offering increased patient convenience. These devices can potentially revolutionize HTN monitoring in various activity and environmental settings that can alter BP, providing a new level of control and understanding. They can also offer a potentially accurate diagnosis of BP phenotypes with worse cardiovascular prognoses, such as nocturnal non-dipping BP, masked hypertension, and pathological BP variability [45].

#### 3.3.1 Oscillometric Wrist Cuff Devices

These cuff-based wrist devices utilize the same oscillometric principle used by traditional sphygmomanometers. Compared to conventional arm-based cuffs, these devices are less uncomfortable for patients. The oscillations are recorded during gradual depressurization. The start of oscillations corresponds with systolic BP, and the oscillations continue below the diastolic BP level. These devices use an algorithm to calculate the BP values based on the input received by the oscillations. These devices are less sensitive to external noise but unreliable during physical activity. The wrist should be placed at the heart level to get an accurate reading as the BP can deviate by 7 mm Hg secondary to hydrostatic pressure if it deviates by 10 cm from the heart [46].

Omron HEM-6410 (HeartGuide-Omron Healthcare Co. Ltd, Kyoto, Japan) is a wearable cuff-based wrist device. It has a highly rigid inflatable belt and is available in two sizes (ZM and ZL). It is user-friendly, has a clocklike display, and can provide multiple measurements in any external environmental condition (stress, work). It can also be programmed to measure during sleep. This device fulfilled the Advancement of Medical Instrumentation/ISO81060-2:2013 criteria when used in a sitting position with the wrist at the heart level [46]. Omron HEM 9600T also fulfilled the Advancement of Medical Instrumentation/ISO81060-2:2013 criteria when used in a sitting position, and the accuracy was maintained in the supine position while the palm was facing downwards [46].

HEM6410T devices were compared with ABPM by simultaneously wearing both devices on the same arm. The mean difference between HTN and ABPM devices was within 10 ± mm Hg, 58.7% in the office and 47.2% outside the office. These differences were not statistically significant in a mixed-effect moderate analysis, suggesting that the readings with these devices are comparable to ABPM BP readings [46].

#### 3.3.2 Cuffless Devices

Cuffless BP devices eliminate the potential errors associated with cuff use, such as patient discomfort, improper cuff size, or placement. Contrary to traditional cuff-based devices, these devices do not directly measure BP. They estimate it by measuring other physiological variables and plugging those variables into an algorithm [47].

#### 3.3.3 Applanation Tonometry Devices

This method utilizes a sensor with a hemispherical air chamber device that directly contacts the arterial wall. This device compresses the artery to make it flat but does not compress completely, leaving it partially open. The mean arterial pressure is measured through oscillometric measurement while the air chamber is decompressed continuously and steadily. The systolic blood pressure (SBP) and diastolic blood pressure (DBP) are derived from the device’s algorithms [45, 46, 47]. The radial artery is ideal for this device as it is underneath the skin against the bone. The sensor should be in a stable and secure position against the artery. The BPRo device (Health STATS Technologies, London, England, and Health STATS International, Singapore) uses the applanation tonometry technique on the radial artery [45, 46, 47]. The measurements are more reliable in sitting and lying down positions than standing. These devices also have reduced accuracy in ambulatory settings, especially in patients with chronic kidney disease, as they tend to have vascular calcification. The measurement should be taken while the wrist is positioned at the heart level to negate the effect of hydrostatic pressure. These devices have been used to monitor nocturnal BP and BP spikes in patients with sleep apnea [45, 46, 47].

#### 3.3.4 Photoplethysmography Devices

PPG evaluates the volumetric changes of tissues secondary to blood flow during the cardiac cycle. PPG devices have a light source and a photodetector that measures blood volume based on the amount of light absorbed. PPG has been used to assess heart rate and pulse ox and detect peripheral venous diseases [45, 46, 47].

Pulse transit time (PTT) is the time the arterial waveform travels from 1 site to a different site (from the heart to a peripheral site). The PTT correlates with arterial stiffness and is inverse to BP. Pulse wave velocity (PWV) can be calculated based on the PTT readings and the distance traveled. BP can then be estimated from PWV by applying Moes-Korteweg and Hughes equations [48]. The PWV will be variable based on the viscoelastic properties of each person’s arterial wall, so individual calibration of the PTT device will be done to a reference standard, a measurement usually done by standard oscillometric BP cuff [48].

PPG devices working based on the PTT principle require 2 PPG sensors. The locations of the 2 PPG sensors can be along the same arterial path, such as the upper arm and fingers, or along different arterial paths, such as the forehead and fingers. BioBeat (BioBeat, Tel Aviv, Israel) has 2 PPG sensors on the back of a wristwatch and measures BP based on the PTT principle. The results of this device’s performance in comparison to the reference standard showed a mean difference of –0.08 with a 95% contrast interval of –7.06 and 6.90 mm Hg for systolic BP and 0.002 with a 95% confidence interval of –6.88 and 6.87 for diastolic BP. The measured bias was higher in patients with hypertension [49].

The pre-ejection period (PEP) is the time interval between left ventricular depolarization and the beginning of the ventricular ejection, and it corresponds to the time of left ventricular contraction against the closed aortic valve. Pulse arrival time (PAT) is equivalent to the sum of PEP and PTT. Devices utilizing PAT principle use the ECG signal to determine R-wave initiation as the proximal site and one peripheral signal site, such as PPG. The ECG data in these devices help calculate the timing of the R-wave, and the PPG sensor provides the volumetric changes during the cardiac cycle. The BP is then estimated based on the measured PAT readings. The devices using PAT make assumptions of the relative contribution of PEP to the PAT, so they are prone to more errors compared to devices that work based on the PTT principle [45, 46, 47].

Recently, BP was estimated using the morphology of the arterial pulse wave through pulse wave analysis (PWA). They could predict SBP, DBP, and pulse pressure by simultaneously measuring facial blood flow in different locations of the face detected through a smartphone. This is an example of contactless optical imaging PPG analysis [45, 46, 47].

### 3.4 Fitness Trackers and Smartwatches

Consumer-grade fitness trackers and smartwatches (commonly called wearables) with cutting-edge optical sensing technology of PPG sensors, which use reflective pulse oximetry to measure vital signs, have been used worldwide since 2010 [50, 51]. PPG absorption is thought to be impacted by skin tones, with darker skin tones interfering with the analysis of heart rate rhythm, leading to undependable readings [52].

These devices are claimed to be at consumer grade to detect a wide variety of simple physiological variables, including heart rate, peripheral blood oxygen saturation, respiratory rate, BP, skin temperature, energy expenditure, and blood oxygen measurement [51, 53, 54, 55]. The accuracy of the measurements from these devices tends to decrease with increased activity levels [56]. Two studies to evaluate the accuracy of heart rate measurements were performed using four different fitness trackers/smartwatches by enrolling healthy volunteers in one study, patients with ischemic heart disease recovering from cardiac procedures in another study showed that the accuracy of all the devices in measuring heart rate decreased with increased exercise intensity [57, 58]. Information related to the data used in calculating energy expenditure is unknown to consumers. Few studies measuring energy expenditure using wearables recommended not to use them for that measurement at sitting or during light to vigorous physical activity [58]. A study done on healthy volunteers in China validated smartwatches to accurately measure maximum oxygen uptake (VO2 max) or the maximum oxygen utilization capacity of an individual, which was used as a tool to detect acute mountain sickness [54].

A case report published in 2022 claimed that healthcare professionals were able to diagnose giant cell arteritis in a patient who presented with an unexplained persistently elevated resting heart rate, which was 50% higher than their baseline (without necessarily having tachycardia) using a smartwatch [59]. The consequences of consumer-grade continuous monitoring through smartwatches and fitness trackers, including but not limited to anxiety, unintended behavioral changes, privacy and security in data handling, and lack of guidance from regulatory bodies in using the data by clinicians, need to be addressed in the future [56].

Specific devices, including sleep analysis, estimation of energy expenditure, changes of physiologic parameters during activity, and pathophysiologic conditions, including atrial fibrillation through heart rhythm and fall detection, also measure complex measurements that combine data from various sensors [52, 53]. These advanced functionalities or complex measurements are often measured differently by different devices, which makes it challenging to compare, leading to insufficient interoperability [53]. A study done by the University of California, San Francisco, showed that for the detection of atrial fibrillation using PPG-based technology devices, conventional analysis, which depends on heart rate series data alone, does not perform with high accuracy compared to a deep learning model implemented in their study using raw PPG-based signal [60].

Smartwatch fitness trackers have recently been designed to be compatible with electronic health records (EHRs), which can help integrate data between EHRs and these devices. Google Fit and Apple HealthKit combine data from multiple health applications and merge it with EHRs, raising concerns about data privacy [56]. A systematic review found that wearing fitness trackers or smart watches has not been shown to reduce mortality, result in weight loss, reduce myocardial ischemic events and strokes, or improve BP or cholesterol levels [61].

Apple Watch, Google, Fitbit, Samsung Galaxy, and Garmin are highly sought after in the field of smartwatches and fitness trackers, with many other brands available in the current consumer market. Apple Watch implements a single-lead ECG to detect atrial fibrillation. Even though the watch has not been studied in patients with a known history of atrial fibrillation, it was approved by the FDA as a class 2 medical device in 2018. Due to the single-lead nature of the Apple Watch ECG, only atrial fibrillation or non-sustained ventricular tachycardia can be recorded currently. The Fitbit Sense model records approximate calories burned, step count, peripheral oxygen saturation, and temperature. It received FDA and conformite europeenne mark approval in Europe for ECG application [52]. Samsung records sleep activity, peripheral oxygen saturation, and calorie tracking, and received FDA approval for ECG monitoring applications. Samsung’s BP tracking is not currently cleared for use in the US [52]. A recent study showed that Apple Watch’s ECG tracings showed acceptable QT-interval measurements, which helped remote QT monitoring in quarantined outpatients taking QT-prolonging medications. However, it was only considered for informational use and not for diagnostic purposes [56]. Apple Watch, Polar Vantage V, Garmin, Fitbit, and Firmware are a few of the devices claimed to measure energy expenditure [55].

## 4. Wearable Technology and Lifestyle Factors

### 4.1 Physical Activity

Physical activity has been established as an integral part of managing primary, secondary, and tertiary cardiovascular disease (CVD) prevention. Regular, well-coordinated, moderate-intensity physical activity reduces the risk of CVD mortality by 30%. It also significantly improves the quality of life, increases life expectancy, and reduces the number of hospitalizations [62, 63, 64, 65, 66]. It helps to maintain an ideal body mass index body mass index (BMI), which reduces the risk of metabolic syndrome, including obesity, high blood pressure, and type 2 diabetes, which are risk factors for cardiovascular disease. It also helps manage hyperlipidemia by increasing high-density lipoprotein (HDL) and decreasing low-density lipoprotein (LDL) and triglycerides. Despite the overwhelming evidence of the benefits of physical activity, approximately 27.5% of adults worldwide are still not physically active, based on data covering most of the world’s population [66, 67, 68]. Even among the patients treated for a cardiovascular event like myocardial infarction, coronary revascularization procedure, or heart failure, only about 30% take part in a physical rehabilitation program for 3 months in a cardiac rehabilitation center [69, 70, 71, 72]. Several studies established that wearable devices might efficiently promote physical activity and sleep quality [73, 74, 75, 76, 77, 78].

Physical fitness wearables, including smartwatches, fitness bands, and even smart clothing, are widely popular devices for individuals looking to stay active, improve their fitness, and track their progress toward set goals. They objectively measure and input real-time feedback on the number of daily steps, total activity time, distance covered, and number of calories burned [65]. They track heart rate in real-time metrics, including resting, active, and exercise heart rate parameters, including vital signs [65, 73, 79, 80, 81, 82]. All activities like running, cycling, and hiking are monitored, giving insights into pace, distance, and elevation in their separate workout modes. The most used wearables include the Apple Watch, Samsung Galaxy, Garmin Forerunner, Fitness Charge, Polar Vantage, Whoop strap, and Xiaomi Mi band, which cover various activity goals. The sensor technologies used are accelerometers to detect movement, gyroscopes to detect orientation, optical heart rate sensors, and GPS to collect data on physical activities. The collected data is integrated with specific partner apps on the smartphone, where detailed reports, set goals, and track progress can be viewed. The data can be shared with healthcare professionals and family members or on social media with a community. These devices provide feedback and motivate users to reach their fitness goals, provide personalized coaching, set and monitor personalized goals, and share within the community while promoting engagement [83, 84]. Besides the above, some devices alert users when they have been sedentary for a long time, prompting them to move. They can provide data for longer time frames and valuable data for healthcare professionals to make shared and informed decision-making [65, 67, 85].

Some wearable devices provide continuous BP monitoring, alerting to early issues, which can also provide data when they see their healthcare professionals by providing a trend analysis. Integration with diet tracking apps allows users to monitor calorie consumption. Some wearable devices can be linked to apps that provide insights into overall calorie and nutrition intake, helping them maintain a balanced diet [86, 87].

### 4.2 Sleep

The Sleep Research Society and the American Academy of Sleep Medicine recommend that adults aged 18–60 regularly obtain seven or more hours of sleep per night to promote optimal health [88]. Adequate, good-quality, regular sleep is essential for maintaining optimal cardiovascular health. Adequate sleep helps control BP, regulates normal heart rate variability, decreases inflammation, and balances stress hormones like cortisol. Chronic insufficient, poor-quality sleep can worsen blood pressure, cause abnormal heart rate variability, inability to cope with stress, and cause increased inflammation, all of which are linked to poor cardiac health. It can also cause insulin resistance, increasing the risk of diabetes and cardiovascular disease [89, 90, 91, 92, 93, 94]. Poor sleep increases the risk of anxiety and depression, which indirectly affects cardiac health [95]. Despite the recommendations of the Sleep Research Society and the American Academy of Sleep Medicine, about 35% of adults in the United States sleep less than 6 hours per night. In some specific groups, such as active military personnel, it even exceeds approximately 40% or more [96, 97, 98, 99]. Several studies established that wearable devices might efficiently promote physical activity and sleep quality [73, 74, 75, 76, 77, 78].

Some common wearable sleep devices include Fitbit, Ocura ring, Whoop strap, Apple watch, and Garmin, which offer a wide range of functional capabilities of sleep analytics [100, 101]. These wearables can range from wristbands and smartwatches to rings and headbands. Some of these devices use third-party apps to have full operational capability. Most sleep wearables use actigraphy, which detects movements by accelerometry and light exposure [102, 103, 104, 105]. Most of these sleep-wearable devices also use a combination of accelerometers to detect movement, optical sensors to estimate heart rate and peripheral oxygen saturation (SpO2), and sometimes temperature sensors to gather data, using algorithms to estimate sleep stages and sleep quality. This data is usually integrated into a smartphone app, providing detailed sleep reports and insights.

**Fig. 3.**
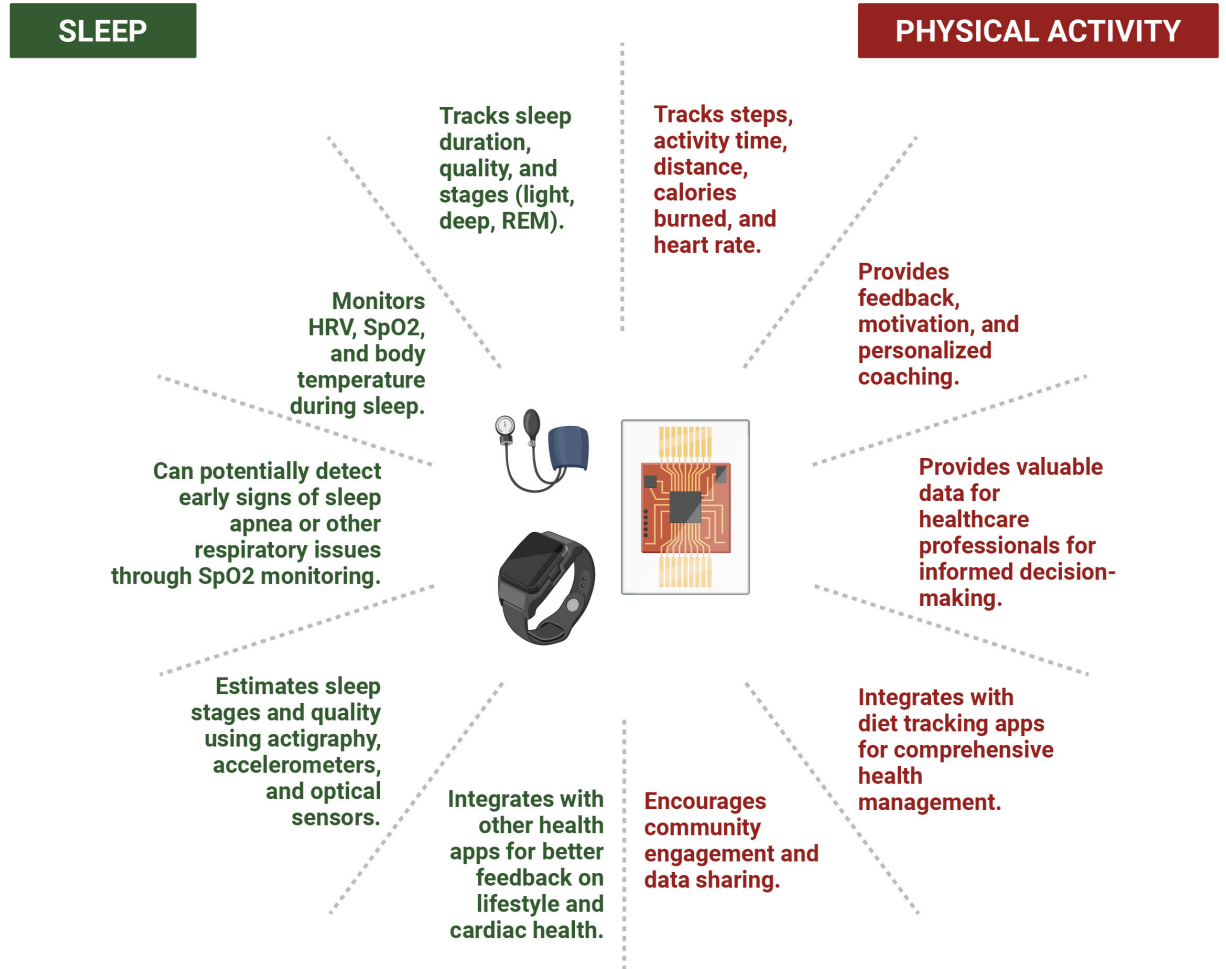
**Impact of Wearable Technology on Sleep & Physical Activity**. REM, rapid eye movement; HRV, heart rate variability; SpO2, peripheral oxygen saturation.

Sleep patterns can be tracked based on sleep duration and quality, as well as light, deep, and rapid eye movement (REM) sleep. Sleep quality can be analyzed by factors like restlessness, wakefulness during the night, and sleep efficiency. They can also monitor heart rate variability (HRV) during sleep. They can also measure the oxygen level in your blood by measuring oxygen saturation (SpO2), which can help diagnose early sleep apnea or other respiratory issues. Some devices can also track your body temperature changes during sleep, which can be related to circadian rhythm issues or illness. Some devices even provide a sleep score based on heart rate, movement, and respiration. This data can provide feedback to improve sleep quality, maintain a regular sleep schedule, and regulate the body’s internal clock. Reducing screen time before bedtime, creating a calming sleep environment, and managing stress by practicing deep breathing and meditation can help promote sleep. This data can be integrated with other health apps while providing better feedback to improve lifestyle and cardiac health [100, 101]. Fig. 3 summarizes the key findings of the impact of wearable technology on sleep and physical activity.

## 5. Key Challenges

### 5.1 Accuracy and Reliability

The accuracy and reliability of wearable devices remain a concern, particularly in consumer-grade models. While medical-grade wearables show high precision in controlled settings, real-world performance is affected by factors such as motion artifacts, skin tone variations, and individual physiological differences. This calls for further research to enhance device accuracy and develop robust algorithms that reduce these confounding variables. Additionally, wearable blood pressure monitors face significant limitations. Cuffless devices do not directly measure blood pressure but estimate it based on surrogate variables. Many of these devices rely on PPG sensors, which have not been validated across diverse patient populations, including individuals with varying skin tones. Moreover, all cuffless devices require calibration against traditional cuff-based monitors, which use oscillometric methods known for their limitations in accuracy. The drift phenomenon, wherein device accuracy deviates from reference standards over time, further complicates long-term reliability and necessitates frequent recalibration.

### 5.2 Data Security and Privacy

As wearable ECG devices become more embedded in cardiovascular care, the protection of sensitive health data is crucial. The vast amount of sensitive health data collected by wearable devices presents a significant challenge regarding security and privacy. Robust measures, including end-to-end encryption, secure and potentially decentralized data storage with granular access controls, and advanced anonymization techniques, are crucial to protect patient confidentiality and prevent misuse. Furthermore, clear and comprehensive regulatory frameworks defining data ownership, access, and sharing, coupled with transparent user consent mechanisms, are essential to ensure ethical data handling practices and foster user trust.

Future efforts must focus on developing and implementing these safeguards, alongside user education on privacy risks and best practices. Emerging technologies like federated learning and homomorphic encryption offer promising avenues for data analysis while preserving privacy. Regular security audits and the establishment of clear accountability for data breaches will also be critical in ensuring the responsible and secure integration of wearable technology in cardiology. Addressing these concerns proactively is paramount to realizing the full potential of wearables without compromising patient privacy and security.

### 5.3 Integration With Healthcare Systems

Seamless integration with existing healthcare systems remains a challenge. Wearable devices generate a continuous stream of data, which can overwhelm the healthcare infrastructure. Efficient data management and utilization require standardized formats and interoperable platforms to facilitate integration with EHRs and clinical workflows. Without these improvements, wearable technology may remain underutilized in routine clinical practice.

### 5.4 Patient Adherence and Compliance

The effectiveness of wearable devices is closely connected to patient adherence. Studies have reported varying levels of compliance, with some registries indicating high median daily usage times (>20 hours), while others, like the VEST trial, have noted lower adherence [36]. Factors influencing compliance include the device’s comfort, user awareness of its clinical significance, and individual lifestyle constraints. Additionally, WCDs may impact mental health, physical comfort, and daily activities, although some studies suggest that quality of life remains stable or even improves for certain patients. Similarly, long-term adherence to wearable blood pressure monitors presents challenges. Motion artifacts during activity can disrupt signal accuracy, and the need for frequent recalibration can be burdensome for patients requiring prolonged use. Moreover, the accuracy of these devices remains uncertain in specific populations, such as those with coronary artery disease, heart failure, pregnancy, larger upper arm circumferences (>50–52 cm), and individuals with cardiac arrhythmias [47].

### 5.5 User Acceptance and Usability

User acceptance and long-term usability are essential for the success of wearable technology. Factors such as device comfort, usability, and cost impact patient adoption and adherence. Future research should emphasize user-centered design and personalized interventions to improve engagement and ensure the sustained use of wearable devices in clinical practice.

## 6. Technical Advancements in Wearable Devices

### 6.1 Recent Innovations and Improvement in Wearable Technology

In recent decades, wearable devices have shown enormous technological advancements due to research in electrochemical and optical biosensors, miniaturization, bioanalysis, material chemistry, including nanomaterials and nanostructures, applied spectroscopy, connectivity, and the application of AI [106, 107, 108, 109, 110]. As discussed in our paper, a plethora of ambulatory wearable electrocardiography devices, including smartphones, smartwatches, and garment-based options, have enabled the monitoring of arrhythmias, notably the real-time and offline detection of atrial fibrillation or atrial flutter [12, 22, 23, 26, 27]. Innovations in BP wearable devices, both in oscillometric wrist cuff devices and cuffless devices utilizing applanation tonometry and PPG, have transformed the measurement and prediction of BP, pulse rate, pulse pressure, and arrhythmias [46, 47].

### 6.2 Integration With Artificial Intelligence and Machine Learning

With the increasing use of wearables and improved data acquisition, an enormous amount of data is available for AI analysis. Wearable devices use neural networks and other ML methods to enhance the diagnostic and predictive capabilities of various cardiovascular diseases [110]. One of the major focuses of many studies is the area of arrhythmia detection, specifically atrial fibrillation [110]. Wearable devices with AI capabilities can provide personalized, proactive healthcare insights, which can help an individual make health changes and monitor the impact of interventions [51]. Analyzing physiological parameters using AI and deep learning models can help to predict cardiovascular diseases such as coronary artery disease, atrial fibrillation, stroke, and hypertension, and prognosticate the severity and mortality of heart failure [20, 111, 112, 113, 114, 115, 116].

## 7. Future Developments

The field of wearable technology in cardiology is rapidly evolving, with several promising avenues. AI, ML, and nanotechnology hold immense potential for enhancing wearables’ diagnostic and predictive capabilities, and the next decade is poised to usher in an era dominated by these technologies. Nanotechnology-enabled biosensors can assist with non-invasive detection of troponin levels, combined with AI models, which can predict the risk of myocardial damage/tissue necrosis [109, 117, 118]. Hybrid devices can integrate the portability of wearable devices and the reliability of implanted devices. Systems such as implantable biosensors within the body and a wearable monitor can provide better patient comfort, reduce side effects, and extend the lifetime of implanted sensors [119]. Another area of advancement is in energy harvesting. Flexible and wearable supercapacitors can provide longer cycle life, higher power density, and faster charging over batteries, while wearable thermoelectric generators (WTEGs) convert body heat into electricity. Piezoelectric nanogenerators (PENGs) convert mechanical stress into electricity, ensuring an endurable energy supply for wearable devices [120, 121, 122].

Additionally, personalized medicine represents a crucial area for future research. Wearable devices provide individualized insights into patient health, enabling tailored interventions and personalized treatment plans, thus enhancing patient care and improving outcomes. Furthermore, long-term studies are necessary to assess wearable technology’s clinical effectiveness and cost-effectiveness in cardiology. Large-scale, randomized controlled trials can deliver robust evidence to support the widespread adoption of wearables in clinical practice.

## 8. Conclusion

Wearable technology’s utility in cardiology has evolved from its historical roots to today’s sophisticated, AI-driven devices. While significant progress has been made, challenges remain in accuracy, reliability, data security, healthcare system integration, patient adherence, and usability. Addressing these challenges is crucial for realizing the full potential of wearables in revolutionizing cardiac care. The future lies in the convergence of AI, machine learning, nanotechnology, and personalized medicine, promising continuous, individualized insights into cardiac health and empowering patients and clinicians alike.

To fully capitalize on these advancements, we must prioritize further research on accuracy and reliability, strengthen data security and privacy measures, enhance healthcare system integration, promote patient adherence, and invest in clinical trials and cost-effectiveness analyses. Collaboration among researchers, clinicians, technology developers, and regulatory bodies is essential to drive innovation and address ethical considerations. By focusing on these key areas, we can unlock the transformative potential of wearable technology, paving the way for a future of proactive, patient-centered cardiac care.

## Abbreviations

ABPM, Ambulatory Blood Pressure Monitoring; ACC, American College of Cardiology; ACS, Acute Coronary Syndrome; AHA, American Heart Association; AI, Artificial Intelligence; BMI, Body Mass Index; CAM, Carnation Ambulatory Monitor; CDC, Centers for Disease Control and Prevention; CMS, Centers for Medicare and Medicaid Services; COVID-19, Coronavirus Disease 2019; CVD, Cardiovascular Disease; DARPA, Defense Advanced Research Projects Agency; DBP, Diastolic Blood Pressure; ECG, Electrocardiogram; EHR, Electronic Health Record; ESC, European Society of Cardiology; FDA, Food and Drug Administration; GDMT, Guideline-Directed Medical Therapy; HBPM, Home Blood Pressure Monitoring; HDL, High-Density Lipoprotein; HRS, Heart Rhythm Society; HTN, Hypertension; ICD, Implantable Cardioverter-Defibrillator; ISO, International Organization for Standardization; LDL, Low-Density Lipoprotein; LV, Left Ventricular; LVEF, Left Ventricular Ejection Fraction; MCOT, Mobile Cardiac Outpatient Telemetry; MI, Myocardial Infarction; ML, Machine Learning; NICM, Non-Ischemic Cardiomyopathy; NR, Not Randomized; PAT, Pulse Arrival Time; PENG, Piezoelectric Nanogenerators; PEP, Pre-Ejection Period; PPG, Photoplethysmography; PTT, Pulse Transit Time; PWA, Pulse Wave Analysis; PWV, Pulse Wave Velocity; QT, QT Interval; REM, Rapid Eye Movement; SBP, Systolic Blood Pressure; SCA, Sudden Cardiac Arrest; SCD, Sudden Cardiac Death; SpO2, Peripheral Oxygen Saturation; USA, United States of America; VA, Ventricular Arrhythmia; VF, Ventricular Fibrillation; VO2 max, Maximum Oxygen Uptake; VT, Ventricular Tachycardia; WCD, Wearable Cardioverter-Defibrillator; WTEG, Wearable Thermoelectric Generators.
